# Heterogeneity in Autonomic Arousal Level in Perseverative Worry: The Role of Cognitive Control and Verbal Thought

**DOI:** 10.3389/fnhum.2017.00108

**Published:** 2017-03-13

**Authors:** Gim Y. Toh, Michael W. Vasey

**Affiliations:** Department of Psychology, The Ohio State University, ColumbusOH, USA

**Keywords:** worry, generalized anxiety disorder, autonomic arousal, verbal worry, cognitive avoidance, effortful control, cognitive control capacity

## Abstract

One puzzle in high worry and generalized anxiety disorder (GAD) is the heterogeneity in the level of autonomic arousal symptoms seen among affected individuals. While current models agree that worry persists, in part, because it fosters avoidance of unpleasant internal experiences, they disagree as to whether worry does so by suppressing activation of autonomic arousal or by fostering persistent autonomic hyperarousal. Our Cognitive Control Model predicts that which pattern of autonomic arousal occurs depends on whether or not a worrier has sufficient cognitive control capacity to worry primarily in a verbal versus imagery-based manner. Because this model has been supported by only one study to date, the present study sought to replicate and extend that study’s findings. Results from an online survey in an unselected sample of over 900 college students provide further support for our model’s central tenet and initial support for its prediction that higher effortful control is associated with a higher percentage of verbal thought during worry. Finally, we report tentative evidence that autonomic arousal symptoms in worry and GAD vary as a function of individual differences in cognitive control capacity because higher capacity is linked to a greater predominance of verbal thought during worry.

## Introduction

Excessive and uncontrollable worry is a common form of perseverative cognition that, at its most severe levels is the hallmark of generalized anxiety disorder (GAD; American Psychiatric Association, 2013). Until recently, such worry was seen as being characterized by low levels of autonomic arousal, a pattern predicted by the Cognitive Avoidance (CognAv) Model of worry (Borkovec et al., 2004). That model posits, in part, that worry is characterized by suppression of fear-provoking images and the autonomic arousal they would typically engender, by shifting to a verbal mode of threat processing. This model is supported by numerous studies finding that worry and GAD are indeed characterized by a lack of elevated autonomic arousal. However, despite such support, a similarly large body of studies shows instead that worry and GAD are characterized by high levels of autonomic arousal. In light of such findings, another model of GAD, the Contrast Avoidance (ContrAv) Model (Newman and Llera, 2011), posits that worry does not serve to limit activation of autonomic arousal but rather to increase and maintain heightened autonomic arousal and negative emotionality more broadly, which permits worriers to avoid unpredictable spikes in such emotional states, which they find aversive. However, whereas the CognAv model cannot account for findings that worry and GAD are characterized by high levels of autonomic arousal, neither can the ContrAv model easily accommodate the opposite pattern. To resolve this conflict, Vasey et al. (2016) recently proposed and tested an integrative model, which posits that only when worriers have sufficient cognitive control capacity to suppress intrusive threatening imagery and shift instead to verbal processing of threat can they avoid the autonomic arousal that such images would otherwise elicit. Absent such capacity, worry will instead be characterized by heightened autonomic arousal. In the initial study, the pattern of results was consistent with this prediction, in both a large, unselected sample and in an analog GAD subsample. Using another sample of over 900 individuals, the current study sought to replicate and extend these findings to show why cognitive control capacity matters.

Prior to the fourth edition of the Diagnostic and Statistical Manual (DSM-IV; American Psychiatric Association, 1994) autonomic arousal symptoms were among the defining features of GAD. Specifically, in the DSM-III-R (American Psychiatric Association, 1987), GAD was defined by unrealistic and excessive worry accompanied by at least 6 of 18 symptoms from three clusters, including *autonomic hyperactivity* (e.g., shortness of breath, accelerated heart rate). However, with the introduction of DSM-IV (American Psychiatric Association, 1994), autonomic arousal symptoms were dropped and remain absent in the DSM-5 (American Psychiatric Association, 2013). This decision was based on the CognAv model and on findings that GAD patients infrequently endorsed these symptoms (e.g., Marten et al., 1993).

There is, in fact, striking heterogeneity in the level of autonomic arousal in worriers and GAD samples (see Vasey et al., 2016 for a review). This is true whether autonomic arousal is measured subjectively (e.g., Marten et al., 1993; Brown and McNiff, 2009) or objectively using heart rate (HR; e.g., Lyonfields et al., 1995; Thayer et al., 1996), non-specific skin conductance responses (NS-SCRs; Andor et al., 2008; Pruneti et al., 2010), and salivary alpha amylase (sAA; Fisher et al., 2010; Fisher and Newman, 2013). There is also evidence of heterogeneity in autonomic arousal in response to emotional provocation whether using threat stimuli (e.g., Grillon, 2008; Pruneti et al., 2010) or worry inductions (e.g., Andor et al., 2008; Llera and Newman, 2014). Neuroimaging studies also reveal such heterogeneity. GAD samples either show significantly less than or do not differ from controls in amygdala activation in response to threat stimuli, while others show significantly higher activation (e.g., Monk et al., 2006, 2008). As a whole, it appears that pathological worry is at times characterized by low levels of autonomic arousal that are not significantly different from levels displayed by healthy controls, and at other times characterized by high levels of autonomic arousal which are not significantly different from that of individuals with panic disorder.

Importantly, several studies have found that worry may blunt autonomic arousal in response to fear-provoking imagery (e.g., Borkovec and Hu, 1990; Borkovec et al., 1993). To the contrary, others have found that a worry period did not suppress autonomic arousal in response to fearful imagery in absolute terms (e.g., Peasley-Miklus and Vrana, 2000; Llera and Newman, 2014). Rather, worry significantly increased HR from baseline, which prevented further increases in HR during presentation of fearful stimuli.

To account for the well-documented heterogeneity in level of autonomic arousal among worriers and individuals with GAD, Vasey et al. (2016) proposed an integrative model. They predicted and found that individual differences in effortful control, a broad self-regulatory construct which encompasses attentional, inhibitory, and activation control (Rothbart, 2007), accounts for this heterogeneity. Specifically, they found that effortful control was negatively associated with autonomic arousal symptoms. Importantly, that negative association was strongest at the highest levels of worry and GAD symptom severity.

Even though worry is generally seen as being associated with deficits in cognitive control resources such as attentional control (e.g., Armstrong et al., 2011; Hirsch and Mathews, 2012), worriers and those with GAD nevertheless vary considerably in their capacity to control their attention. As reviewed in Vasey et al. (2016), worriers and individuals with GAD vary in effortful control and related constructs when measured using self-report (e.g., Armstrong et al., 2011; Rosellini and Brown, 2011), behavioral measures (e.g., Derryberry and Reed, 2002; Olatunji et al., 2011), neuroimaging (e.g., Etkin et al., 2009; Price et al., 2011), and a physiological index of capacity for top–down control (i.e., resting heart rate variability [HRV; see Thayer et al., 2009 for a review; Brosschot et al., 2007; Aldao et al., 2012]).

Such individual differences in effortful control among worriers and GAD samples are especially important given that there appears to be a negative relationship between executive function and autonomic arousal symptoms (Beaudreau and O’Hara, 2009; Etkin et al., 2009; Richey et al., 2012). For example, in addition to finding an atypical pattern of functional connectivity between the amygdala and the dorsolateral prefrontal cortex (dlPFC) in GAD patients, Etkin et al. (2009) found that the strength of that connectivity was significantly *negatively* associated with scores on the Beck Anxiety Inventory (BAI; Beck and Steer, 1990), which is predominantly a measure of autonomic arousal symptoms (Leyfer et al., 2006). Consequently, they concluded that at least some GAD patients exhibit habitual engagement of an executive control system to regulate autonomic arousal symptoms.

However, the demonstration that effortful control moderates the association between worry/GAD symptom severity and autonomic arousal does not elucidate the mechanism by which it does so. Vasey et al. (2016) proposed that a closer examination of the CognAv model reveals a mechanism by which individual differences in cognitive control capacity can impact the level of autonomic arousal triggered by worry. As stated by Borkovec et al. (2004, p. 83), “...when aversive images occur in the process of worry...the shifting of attention to [verbal] worrisome thinking upon each occurrence...results in escape from or avoidance of the somatic element of the fear response...,” suggesting that heterogeneity in autonomic arousal symptoms may depend on the extent to which verbal or imaginal processing predominates during worry.

The proposed mechanism of the CognAv model is supported by several points. First, visual images rather than verbal thoughts of feared stimuli are more likely to activate autonomic arousal responses (e.g., Tucker and Newman, 1981; Vrana et al., 1986). Additionally, studies have found that people spontaneously shift from imagery to verbalization to reduce autonomic arousal when processing aversive material (Tucker and Newman, 1981; Borkovec et al., 1998). Moreover, verbal thoughts predominate over imagery during worry (Borkovec and Lyonfields, 1993), especially in GAD patients (Hirsch and Mathews, 2012). Indeed, worry is characterized by a predominance of left-frontal cortical activity (e.g., Wu et al., 1991; Hofmann et al., 2005), which has been linked to verbal thought (Tucker, 1981; Pinker, 1994). Additionally, sustaining a verbal linguistic mode of worry is more taxing on working memory resources than worrying in an imaginal form (Leigh and Hirsch, 2011), suggesting that high effortful control capacity may be instrumental in maintaining a predominantly verbal form of worry. Nevertheless, despite the evidence that verbal processing predominates over imagery during worry, others have found otherwise (e.g., Borkovec et al., 1993; Stapinski et al., 2010). Furthermore, it appears that differences in the extent to which verbal worry predominates can account for differences in autonomic arousal (Borkovec et al., 1993). For example, Borkovec et al. (1993) found that percentage of verbal worry reported by participants was significantly negatively correlated with HR response whereas in the relaxation condition, percentage of imagery was significantly positively correlated with HR response. Thus, it appears that the presence of autonomic arousal symptoms depends on the extent to which verbal or imaginal processing predominates during worry, which in turn depends on the worrier’s cognitive capacity to emphasize the former mode of processing over the latter.

The current study was an attempt to replicate Vasey et al.’s (2016) findings about the role of cognitive control capacity (specifically effortful control) in the heterogeneity of autonomic arousal symptoms in worry and GAD, especially when worry is pathological. Second, the current study extended prior work by testing the second major aspect of the model. No previous study has tested our model’s prediction that individual differences in effortful control capacity moderate the association between worry/GAD symptoms and the extent to which worry involves verbal thought. Specifically, the current study employed self-report questionnaires in an unselected sample to test the following predictions:

(1) Effortful control will moderate the positive association between worry/GAD symptoms and autonomic arousal symptoms, such that it is strongest when effortful control is low and weakest when effortful control is high.
  (a) In an analog GAD subsample, effortful control will be significantly negatively correlated with autonomic arousal.
(2) Effortful control will moderate the association between worry/GAD symptoms and candidate mediators, including (a) verbal thoughts during worry, (b) imagery during worry, and (c) efforts to transform images into thoughts, such that higher effortful control will predict more verbal thoughts and efforts to transform images into thoughts and less imagery during worry.
(3) If effortful control emerges as a significant moderator of the association between worry/GAD symptom severity and any of the candidate mediators, we expect that moderated mediation (Hayes, 2013) will be observed, such that the indirect path from worry/GAD symptoms to autonomic arousal symptoms through the mediator will vary significantly as a function of effortful control.

## Materials and Methods

### Participants and Procedure

The sample comprised 990 undergraduates at a large Midwestern university (mean age = 18.8 [*SD* = 1.4]; 54.4% female, 80% White, 4% African American, 9% Asian, 3% Latino/Latina, 1% Native American, and 4% mixed ethnic heritage) who received partial course credit for participation. Participants received a broad description about a 30-min online set of questionnaires related to worry and psychological adjustment. Participants were informed that they were free to decline to participate, stop at any point during the questionnaire, or decline to answer any question without penalty. De-identified responses were collected using Surveymonkey, a secure, web-based data collection service.

### Measures

#### The Generalized Anxiety Disorder Questionnaire IV (GADQ-IV)

The Generalized Anxiety Disorder Questionnaire IV (GADQ-IV) (Newman et al., 2002) is a self-report questionnaire designed as a screening measure that captures the full diagnostic criteria for GAD according to the DSM-IV. The GADQ-IV has good test–retest reliability, convergent and discriminant validity, and good agreement with diagnostic interviews (Newman et al., 2002; Moore et al., 2014). We used the GADQ-IV as a measure of GAD symptom severity and scored it without the skip structure as reported in Vasey et al. (2016) and as recommended by Rodebaugh et al. (2008). As shown in **Table 1**, the internal consistency of the GADQ-IV was high in the present study.

**Table 1:** Descriptive statistics.

		Full sample *N* = 926		GAD sample (12.9%) *N* = 120	
	Cronbach’s alpha	*M*	*SD*	*M*	*SD*
GADQ-IV	0.85	5.5	3.2	10.7	0.75
WAQ	0.92	36.8	17.2	58.7	11.9
PSWQ	0.93	49.9	13.6	67.1	10.0
DASS-Anxiety	0.87	6.6	6.2	26.9	6.9
DASS-Depression	0.93	7.9	8.1	30.3	9.9
DASS-Stress	0.92	11.6	8.2	35.7	8.1
EC	0.82	45.3	6.8	42.2	7.2
CAQ-Transform	0.84	12.2	4.5	14.6	4.4
Percentage of thoughts	–	64.3	26.5	70.8	19.2
Percentage of images	–	25.7	19.7	25.4	16.7

*GADQ-IV, Generalized Anxiety Disorder Questionnaire-IV; WAQ, Worry and Anxiety Questionnaire; PSWQ, Penn State Worry Questionnaire; DASS, Depression, Anxiety, and Stress Scale; EC, Effortful Control Scale; CAQ-Transform, Cognitive Avoidance Questionnaire – Transformation of Images into Thoughts subscale.*

#### Worry and Anxiety Questionnaire (WAQ)

The Worry and Anxiety Questionnaire (WAQ) (Dugas et al., 2001) consists of 11 items covering DSM-IV diagnostic criteria for GAD. The WAQ has satisfactory test–retest reliability and good known-groups validity (Dugas et al., 2001). As shown in **Table 1**, the internal consistency of the WAQ was high in the present study.

#### Penn State Worry Questionnaire (PSWQ)

The Penn State Worry Questionnaire (PSWQ) (Meyer et al., 1990) is a self-report measure of pathological worry, which comprises 16 items rated on a Likert scale ranging from 1 (Not at all typical) to 5 (Very typical). This scale has excellent psychometric properties (Meyer et al., 1990). As shown in **Table 1**, the internal consistency of the PSWQ was high in the present study.

#### The Depression, Anxiety, and Stress Scales (DASS)

The Depression, Anxiety, and Stress Scales (DASS) (Lovibond and Lovibond, 1995) is a 42 items questionnaire comprising three 14 item subscales measuring symptoms of anxiety (DASS-A), stress and depression. Participants rate each item on a four-point Likert scale ranging from 0 (Did not apply to me at all) to 3 (Applied to me very much, or most of the time) regarding how much the item applied to them over the past week. The current study focused on the DASS-A, which predominantly measures autonomic arousal symptoms (Brown et al., 1998). The DASS-A has good psychometric properties (Lovibond and Lovibond, 1995) and, as shown in **Table 1**, its internal consistency was high in the current sample.

#### Effortful Control Scale (ECS)

The Effortful Control Scale (ECS) (Lonigan and Phillips, 1998, Unpublished) comprises 24 items rated on a five-point scale from 1 (Not at all) to 5 (Very much) with regard to how much each describes the individual most of the time. The ECS yields two subscale scores reflecting Persistence/Low Distractibility (ECS-PLD; 12 items) and Impulsivity (ECS-I; 12 items). In this study we focused on the ECS-PLD subscale, which focuses on attention control and the capacity to persist in activities despite reactive motivation to avoid. Example items from this subscale include, “It’s very hard for me to concentrate on a difficult task when there are noises around (R)” and “I can quickly switch from one task to another.” The measure has good psychometric properties in college samples (Vasey, 2014, Unpublished) and, as shown in **Table 1**, it had high internal consistency in the current study. In an independent unselected college sample of over 700 subjects, the ECS-PLD subscale correlated strongly with the Adult Temperament Questionnaire EC Scale (*r* = 0.61, *p* < 0.0001). ECS-PLD scores also behave in the expected fashion in other contexts. For example, Vasey et al. (2014) used the ECS-PLD subscale to demonstrate that individual differences in self-regulatory capacity moderate the associations of negative and positive emotionality with depressive symptoms. The ECS-PLD subscale is hereinafter labeled EC.

#### Percentage of Thoughts and Images

These constructs were assessed with two open-ended questions. This self-report method was used successfully to assess the percentage of thoughts and images during worry in a large unselected sample (Freeston et al., 1996), and the findings were consistent with percentages found in thought sampling studies (e.g., Borkovec and Inz, 1990). To ensure that participants understood the question, they were first given an explanation of imagery versus verbal thought: “Images are when you are generating a picture in your mind and really concentrating on what you can see, feel, smell, hear, and taste in the image. Images are often very vivid because you’re tuning into all of your senses. Verbal thoughts are when you’re thinking using words and silently talking to yourself, like an internal running commentary or dialog. When you’re thinking in verbal thoughts you are thinking in words and sentences” (Leigh and Hirsch, 2011). Participants were then asked to report the percentage of time spent in thoughts and images during worry. The questions about images and thoughts were as follows: “What percentage of your worry is made up of thoughts?” and “What percentage of your worry is made up of images?”

#### Cognitive Avoidance Questionnaire (CAQ)

The Cognitive Avoidance Questionnaire (CAQ) (Gosselin et al., 2002) contains 25 items assessing efforts to use cognitive avoidance strategies such as thought replacement, thought suppression, and distraction. This scale has been validated and translated into English (Sexton and Dugas, 2008). The CAQ has very good test–retest reliability over 4 weeks, *r* = 0.81, and shows evidence of convergent validity and criterion-related validity (Gosselin et al., 2002). In the current study, we used the Transformation of Images into Verbal Thought subscale (CAQ-Transform), which measures efforts to transform images into thoughts. This subscale has a good psychometric properties and, as shown in **Table 1**, it had good internal consistency in the current sample.

### Data Analytic Strategy

Study hypotheses were tested through multiple linear regression (MLR) analyses. All non-dichotomous predictors were mean-centered by z-transformation in these analyses (Aiken and West, 1991). All product terms used in these analyses to test interactions were computed from the standardized predictor variables. Additionally, all dependent variables (i.e., DASS-A and CAQ-transform) except those with readily interpretable scales (i.e., percentage of thoughts and images) were also standardized. Regression diagnostics were examined for each model to determine if extreme data points were present that might be exerting excessive influence on overall model fit or on individual regression coefficients. Specifically, for each model we examined the standardized DFFITS and DFBETA values using ±1.0 as a cutoff (Cohen et al., 2002). No high influence cases were identified in any analysis.

Significant interactions were probed using PROCESS, a computational tool for SPSS (Hayes, 2013)^1^. Specifically, PROCESS utilizes the Johnson–Neyman technique for deriving regions of significance, which identify the range of values of the moderator where the simple slope of the predictor is significant (Preacher et al., 2007). In this manner we reported the regions of significance and illustrated interactions by depicting the predictors’ effect on the dependent variable at high (90th percentile) and low (10th percentile) levels of the moderator. Because we were primarily interested in those with high worry/GAD symptom severity, we also tested EC’s effect on the dependent variable at high (90th percentile) levels of worry/GAD symptom severity.

Statistical power to detect an interaction is a function of the variability in the product term representing that interaction (McClelland and Judd, 1993). Because the WAQ measures GAD symptoms using a Likert scale rather than the mostly dichotomous items on the GADQ-IV, we expected that the product term representing its interaction with EC would have more variability than the product terms involving the GADQ-IV. For that reason, we chose it as our primary predictor. Ancillary tests based on the GADQ-IV and PSWQ are reported in the Supplementary Material. Consistent with this rationale, the standard deviation for the WAQ × EC interaction (*SD* = 1.09) was descriptively larger than that of the GADQ-IV or PSWQ × EC interaction (*SD* = 1.06 and 1.02, respectively).

**FIGURE 1:**
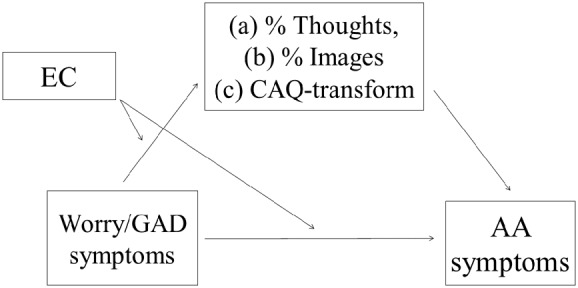
**Conceptual model for tests of moderated mediation**.

Finally, we examined EC as a moderator for the indirect path between worry/GAD symptom severity and autonomic arousal symptoms for any candidate mediator that was significantly predicted by the worry/GAD symptom × EC interaction. Specifically, PROCESS (Model 8) was used to conduct tests of moderated mediation as depicted in **Figure 1**. A bootstrapping approach was used in these tests as recommended by Preacher et al. (2007) because it avoids the assumption of normally distributed products of the coefficients. Specifically, using PROCESS, we conducted bootstrapped (5000 resamples) tests of each mediator at the 10th, 25th, 50th, 75th, and 90th percentiles of the moderator. Furthermore, because we were most interested in individuals with high levels of worry/GAD symptoms, we also examined EC’s indirect effect on autonomic arousal symptoms via the mediator at high (90th percentile) levels of worry/GAD symptom severity. These tests should be interpreted with caution due to the cross-sectional nature of the current study. Recent simulation studies show that cross-sectional tests of mediation can produce biased estimates of the indirect effect (e.g., Maxwell et al., 2011). However, like Hayes and Rockwood (2016), we believe that such tests can still be useful in theory testing. In the present case, we believe such a test is a reasonable, albeit tentative, initial test of the plausibility of our model. At any given point in time, high trait worriers will engage in more verbal thought during worry when they have high versus low levels of trait effortful control. To the extent that they do so, such high effortful control worriers should show less activation of autonomic arousal than those lower in effortful control.

Additionally, to determine if our model holds even at very high levels of worry/GAD symptom severity, we also tested our first hypothesis in an analog GAD sample. That is, among individuals with high levels of GAD symptom severity, EC should be significantly negatively correlated with autonomic arousal. In this context it is important to note that the viability of our model does *not* require that the GAD Symptom Severity × EC interaction be significant in such a subsample. Although we found that interaction to be significant in the analog GAD subsample in our original study (Vasey et al., 2016), we have since realized that result was surprising. That reflects the fact that as one constrains the range of GAD symptom severity, one reduces variance in the product term representing the GAD Symptom Severity × EC interaction. That, in turn, reduces statistical power to find an effect of the interaction (see McClelland and Judd, 1993). To be clear, that reduction in power is above and beyond any reduction in power associated with the smaller sample size of the analog GAD subsample. In essence, as one constrains the range of GAD symptom severity to very high levels, the interaction term becomes redundant with the EC main effect because it ceases to vary much beyond the variance in EC because the range of GAD symptom severity has been severely restricted. Instead, at high levels of GAD symptom severity, our model is most powerfully evaluated in terms of the magnitude and significance of the EC main effect (controlling for remaining variance in GAD symptom severity). Provided that main effect is significantly negative, our model would be supported. With that in mind we tested EC’s effect predicting DASS-A scores while controlling for WAQ scores in our analog GAD group.

There are several approaches for identifying those likely to meet criteria for GAD on the GADQ-IV (Moore et al., 2014). In the current study, the analog GAD subsample included all participants who met DSM-IV criteria based on the GADQ-IV.^2^ Our only additional requirement was that their score on the GADQ-IV ≥ 9, a cutoff found by Newman et al. (2002) to yield 97% specificity and a false positive rate of only 4% in a similar college sample. That approach yielded a group of 120 cases (72.5% female). As shown in **Table 1**, this approach resulted in an analog GAD group characterized by very high levels of worry and GAD symptoms. Specifically, this group had a mean score of 67.09 (*SD* = 9.96) on the PSWQ, higher than the mean for analog GAD samples (i.e., 63.58) and comparable to clinical GAD samples (i.e., 67.16) as reported by Startup and Erickson (2006).

Second, we also tested our first hypothesis using an analog GAD group drawn from a large sample created by combining the current sample with the sample from the Vasey et al. (2016) study. From the resulting group of 2249 cases we chose a subsample of cases based on the GADQ-IV score^3^ (scored following the original scoring approach of Newman et al., 2002). Specifically, we identified the GADQ-IV score defining approximately the top 5% of cases.^4^ A cutoff GADQ-IV score of 11.0 identified 5.3% of cases (*N* = 119; *n* = 73 [5.5%] from Vasey et al. (2016) and *n* = 46 [5.0%] from the current sample). All but two members of this group (i.e., 98.3%) met DSM-IV criteria based on the GADQ-IV. They had a mean score of 69.08 (*SD* = 9.05) on the PSWQ – a value which is significantly above the average PSWQ score across studies of analog GAD samples (*p* < 0.001) and comparable to clinical GAD samples (see Startup and Erickson, 2006). Additionally, their mean score on the GADQ-IV was 11.52 (*SD* = 0.48). That value is significantly higher than in our previous analog GAD sample (*p* < 0.001) and roughly comparable to most other analog GAD samples (e.g., Fisher et al., 2010; Fisher and Newman, 2013). Finally, PSWQ, WAQ, GADQ-IV, EC, and DASS-A scores did not differ significantly by sample (*p*s > 0.12), suggesting that both subsamples were comparable in severity.

## Results

### Preliminary Analyses

Data from 926 of 990 participants are reported (93.5% of the original sample, 55% female). Data from the other 64 participants were excluded because they exhibited suspicious patterns of responding (i.e., excessive missing data [>50% of all questionnaires], nonsensical values, repeat entries, or a repetitive pattern of responding). For the remaining 926 participants, incomplete items and missing data were handled using a two-step process. First, for participants with incomplete data who had less than 50% missing items within a questionnaire, their individual means were used to compute their total score. Individual mean substitution when internal consistency of a questionnaire is strong does not produce substantial bias and is more desirable than discarding individuals from the dataset (Osbourne, 2013). Next, the expectation-maximization (EM) method was used to impute missing values for single-item questions as well as total scores for questionnaires (participants missing more than 50% of items within the questionnaires; 9 cases [1.0%] had 1 missing scale score, 7 cases [0.8%] had 2 missing scale scores, and 2 [0.2%] had 3 missing scale scores). Data were missing completely at random (Little’s MCAR test: *p* = 0.353) and the group with missing values did not differ significantly from the group with complete data on any variable^5^.

### Descriptive Statistics

Means, standard deviations (*SD*), and internal consistency reliabilities for all measures (i.e., Cronbach’s coefficient alpha) are presented in **Table 1**. Zero-order correlations are presented in **Table 2**. Several correlations were particularly noteworthy. As expected, GADQ-IV, WAQ, and PSWQ scores were significantly negatively associated with EC scores but only moderately so (*r* = -0.32, -0.38, and -0.24, respectively). Next, EC scores were significantly negatively associated with DASS-A scores (*r* = -0.44). Finally, as expected, GADQ-IV, WAQ, and PSWQ scores were significantly positively associated with percentage of thoughts (*r* = 0.15, 0.16, and 0.17 respectively).

**Table 2:** Zero-order correlations.

	Sex	GADQ- IV	WAQ	PSWQ	DASS-A	DASS-D	DASS-S	EC	% Thoughts	% Images
GADQ-IV	0.27^∗∗∗^									
WAQ	0.24^∗∗∗^	0.77^∗∗∗^								
PSWQ	0.32^∗∗∗^	0.75^∗∗∗^	0.68^∗∗∗^							
DASS-Anxiety	0.03	0.54^∗∗∗^	0.57^∗∗∗^	0.42^∗∗∗^						
DASS-Depression	0.02	0.53^∗∗∗^	0.56^∗∗∗^	0.42^∗∗∗^	0.70^∗∗∗^					
DASS-Stress	0.15^∗∗∗^	0.66^∗∗∗^	0.70^∗∗∗^	0.60^∗∗∗^	0.74^∗∗∗^	0.70^∗∗∗^				
EC	0.02	-0.32^∗∗∗^	-0.38^∗∗∗^	-0.24^∗∗∗^	-0.44^∗∗∗^	-0.44^∗∗∗^	-0.39^∗∗∗^			
% Thoughts	0.10^∗∗^	0.15^∗∗∗^	0.16^∗∗∗^	0.17^∗∗∗^	0.02	0.09^∗∗^	0.12^∗∗∗^	-0.02		
% Images	-0.04	0.03	0.03	-0.03	0.08^∗^	0.03	0.03	-0.04	-0.41^∗∗∗^	
CAQ-Transform	0.06^†^	0.27^∗∗∗^	-0.33^∗∗∗^	0.22^∗∗∗^	0.36^∗∗∗^	0.29^∗∗∗^	0.30^∗∗∗^	-0.26^∗∗∗^	-0.05	0.16^∗∗∗^

*N = 926. ^∗∗∗^*p* < 0.001; ^∗∗^*p* < 0.01; ^∗^*p* < 0.05; ^†^*p* < 0.10. GADQ-IV, Generalized Anxiety Disorder Questionnaire-IV; WAQ, Worry and Anxiety Questionnaire; PSWQ, Penn State Worry Questionnaire; DASS, Depression, Anxiety, and Stress Scale; EC, Effortful Control Scale; CAQ-Transform, Cognitive Avoidance Questionnaire – Transformation of Images into Thoughts subscale.*

### Did Effortful Control Interact with GAD Symptom Severity to Predict Autonomic Arousal?^6^

**FIGURE 2:**
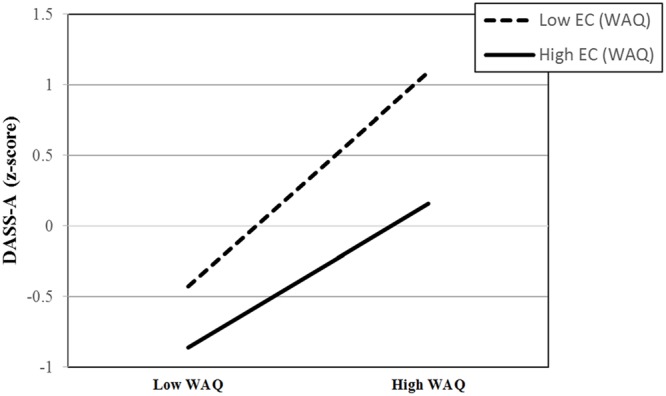
**Graph of the WAQ × EC interaction predicting DASS-A at the 10th and 90th percentile of WAQ and EC**.

**Table 3** shows that on average DASS-A scores were significantly positively predicted by the WAQ and significantly negatively predicted by EC. The WAQ × EC interaction was also significant. WAQ scores significantly positively predicted DASS-A scores across all levels of EC. However, that association was stronger when EC was low (*B* = 0.57, *p* < 0.001) versus high (*B* = 0.38, *p* < 0.001; see **Figure 2**). From the reverse perspective, EC scores significantly negatively predicted DASS-A scores when the WAQ score was high (*B* = -0.35, *p* < 0.001).

**Table 3:** Regression model testing WAQ × EC predicting DASS-Anxiety, percentage of imagery, and CAQ-transform.

	*DV:DASS-A*			*DV:% Imagery*			*DV:CAQ-transform*		
Step/variable	R^2^/B	ΔR^2^/SE	*sr*	R^2^/B	ΔR^2^/SE	*sr*	R^2^/B	ΔR^2^/SE	*sr*
*Step 1*	0.383ˆ***	-		0.002	-		0.130^∗∗∗^	-	
*Step 2*	0.389ˆ**	0.006^∗∗∗^		0.003	0.001		0.132	0.002	
Intercept	-0.01	0.03		25.69^∗∗∗^	0.65		0.01	0.03	
WAQ	0.47ˆ***	0.03	0.44^∗∗∗^	0.37	0.70	0.02	0.27^∗∗∗^	0.03	0.25^∗∗∗^
EC	-0.26ˆ***	0.03	-0.23^∗∗∗^	-0.73	0.70	-0.03	-0.16^∗∗∗^	0.03	0.14^∗∗∗^
WAQ × EC	-0.07ˆ**	0.02	-0.08^∗∗^	0.58	0.60	0.03	0.04	0.03	0.05

*N = 926. ^∗∗∗^*p* < 0.001; ^∗∗^*p* < 0.01. WAQ, Worry and Anxiety Questionnaire; DASS-A, Depression, Anxiety, and Stress Scale – Anxiety subscale; EC, Effortful Control Scale; CAQ-Transform, Cognitive Avoidance Questionnaire –Transformation of Images into Thoughts subscale.*

### Was Effortful Control Negatively Correlated with Autonomic Arousal in the Analog GAD Groups?

#### Current Sample

Consistent with expectation, results showed that after controlling for WAQ scores, the EC main effect was significantly negative (semi-partial *r* = -0.23, *p* = 0.008) in the analog GAD group.

#### Combined Sample

In the analog GAD group from the combined sample, results showed after controlling for GADQ-IV score, the EC main effect was significantly negative (semi-partial *r* = -0.31, *p* < 0.001). Furthermore, this association was not significantly moderated by sample (*p* = 0.69). Thus, EC behaved in similar fashion at high levels of GAD symptoms in both samples.

### Did Effortful Control Interact with GAD Symptom Severity to Predict Percentage of Thoughts?^7^

**FIGURE 3:**
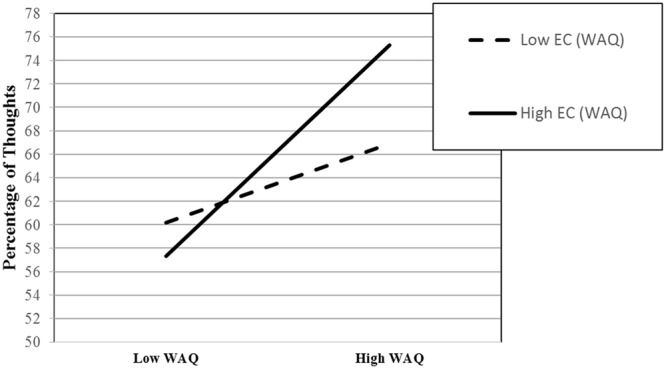
**Graph of the WAQ × EC interaction predicting percentage of thoughts at the 10th and 90th percentile of WAQ and EC**.

**Table 4** shows on average that percentage of thoughts was significantly positively predicted by the WAQ but not significantly negatively predicted by EC. However, the WAQ × EC interaction was also significant. WAQ scores significantly positively predicted percentage of thoughts when EC > -0.126 *SD*. Thus, that association was significant when EC was high (*B* = 6.73, *p* < 0.001) versus low (*B* = 2.51, *p* = 0.081; see **Figure 3**). From the reverse perspective, EC significantly positively predicted percentage of thoughts when the WAQ was high (*B* = 3.18, *p* < 0.019).

**Table 4:** Moderated mediation results involving WAQ × EC predicting DASS-A through percentage of thoughts.

	*Predictor: WAQ*		
Step/variable	R^2^/B	*SE*	*sr*
**DV: Percentage of thoughts**	0.0317^∗∗∗^		
Intercept	64.30	0.86	
WAQ	4.71^∗∗∗^	0.93	0.16^∗∗∗^
EC	1.21	0.93	0.04
WAQ × EC	1.58^∗^	0.79	0.07^∗^
**DV: DASS-A**	0.393^∗∗∗^		
Intercept	0.13^†^	0.07	
Percentage of thoughts	-0.002^∗^	0.001	-0.06^∗^
WAQ	0.48^∗∗∗^	0.03	0.44^∗∗∗^
EC	-0.25^∗∗∗^	0.03	-0.24^∗∗∗^
WAQ × EC	-0.07^∗∗^	0.02	-0.07^∗∗^

*N = 926. ^∗∗∗^*p* < 0.001; ^∗∗^*p* < 0.01; ^∗^*p* < 0.05; ^†^*p* < 0.10. WAQ, Worry and Anxiety Questionnaire; DASS-A, Depression, Anxiety, and Stress Scale – Anxiety subscale; EC, Effortful Control Scale.*

### Did Effortful Control Interact with GAD Symptom Severity to Predict Percentage of Imagery?^8^

As shown in **Table 3**, there were no significant effects of WAQ, EC, or their interaction predicting percentage of imagery during worry.

### Did Effortful Control Interact with GAD Symptom Severity to Predict Efforts to Transform Images into Thoughts?^9^

As **Table 3** shows, on average CAQ-transform scores were significantly positively predicted by WAQ (*B* = 0.27, *p* < 0.001) and significantly negatively predicted by EC (*B* = -0.16, *p* < 0.001). However, the WAQ × EC interaction did not achieve significance (*B* = 0.04, *p* = 0.143).

### Did Percentage of Verbal Thoughts Mediate the Association between GAD Symptom Severity and Autonomic Arousal Conditional upon Level of Effortful Control?^10,^^11^

Given that EC moderated the link between the WAQ and percentage of thoughts, we also examined whether this effect was related to autonomic arousal symptoms. Specifically, we used a moderated mediation model to test whether the relationship between WAQ and DASS-A was mediated by percentage of thoughts but conditional upon level of EC. Based on the MLR model shown in **Table 5**, results supported significant moderated mediation predicting DASS-A (index of moderated mediation^12^ = -0.0038; *SE* = 0.0028; lower limit of CI [LLCI] = -0.0117; upper limit of CI [ULCI] = -0.0001), with the effect being significantly stronger at high versus low levels of EC. As shown in **Table 5**, the pattern was as expected, such that at high EC, WAQ predicted a higher percentage of thoughts, which in turn predicted lower DASS-A scores. As predicted, that indirect path weakened at lower levels of EC. Importantly, as reported in **Table 5** and as expected, viewed from the reverse perspective, when WAQ was high (i.e., 90th percentile), there was a negative indirect effect of EC on DASS-A by virtue of its association with higher percentage of thought^13^.

**Table 5:** Bootstrapped estimates of the conditional indirect paths for the effect of WAQ and EC on DASS-A through percentage of thoughts.

	Indirect effect	Bootstrapped *SE*	Bootstrapped LLCI	Bootstrapped ULCI
Indirect effects of WAQ at varying levels of EC				
10th	**-0.0061**	**0.0046**	**-0.0185**	**-0.0002**
25th	**-0.0089**	**0.0047**	**-0.0209**	**-0.0017**
50th	**-0.0117**	**0.0056**	**-0.0248**	**-0.0025**
75th	**-0.0140**	**0.0067**	**-0.0297**	**-0.0030**
90th	**-0.0162**	**0.0080**	**-0.0360**	**-0.0035**
Indirect effect of EC at 90th percentile of WAQ	**-0.0077**	**0.004**	**-0.0195**	**-0.0011**

*N = 926. Bootstrapped estimates are based on 5,000 samples. Significant effects appear in bold. LLCI, lower limit of confident interval; ULCI, upper limit of confident interval. WAQ, Worry and Anxiety Questionnaire; EC, Effortful Control Scale.*

## Discussion

This study extended work on the Cognitive Control Model of pathological worry in two significant ways. First, it offers additional evidence by providing a replication of Vasey et al.’s (2016) findings that cognitive control capacity acts as a moderator to explain the heterogeneity in level of autonomic arousal associated with worry and GAD, especially in the pathological worry range. As expected, the current results provide support for this integrated model in self-reported data from an unselected sample as well as in two overlapping analog GAD subsamples. Specifically, in the current unselected sample we found that individual differences in effortful control moderated the link between worry/GAD symptom severity and autonomic arousal symptoms such that this link is strongest when effortful control was low and weakest when effortful control was high. That analysis also showed that the effortful control was most strongly negatively correlated with autonomic arousal symptoms among those highest in GAD symptoms (i.e., those at or above the 90th percentile). Furthermore, results from both analog subsamples lend confidence to the conclusion that this negative association between effortful control and autonomic arousal occurs even at very high levels of GAD symptoms.

The strongest evidence for this comes from a subsample of the most severe worriers (i.e., the top 5.3% of scorers on the GADQ-IV) from over 2200 members of a sample combining the current sample with that from our original study (Vasey et al., 2016). Even in that analog GAD subsample EC was significantly negatively correlated with autonomic arousal symptoms (semi-partial *r* = -0.31), which bolsters confidence that such estimates of the effect (i.e., simple slope) of EC at high levels of GAD symptoms from large unselected samples are likely to generalize to those with pathological levels of worry and GAD symptoms.

The second goal of this study was to extend our previous findings by testing the second major aspect of the Cognitive Control Model. Specifically, no previous study has tested the model’s prediction that individual differences in effortful control capacity moderate the association between worry/GAD symptoms and the extent to which worry involves verbal thought. Consistent with that prediction, individual differences in effortful control interacted significantly with GAD symptom severity to predict percentage of verbal thoughts during worry. Most importantly, effortful control was significantly negatively associated with percentage of verbal thoughts when GAD symptoms were high. Although the variance accounted for by this regression model was small, it is important to remember that the dependent variable was a single, retrospective questionnaire item. As such, its reliability is undoubtedly limited.

Finally, the moderated mediation analysis further offers tentative support for the plausibility of our model. Specifically, GAD symptom severity predicted higher percentage of thoughts during worry which in turn predicted lower autonomic arousal symptoms when effortful control was high versus low. This effect is perhaps clearer when viewed from the perspective of the indirect effect of effortful control on autonomic arousal symptoms at high (i.e., 90th percentile) GAD symptoms. Specifically, the indirect effect was significantly negative, by way of effortful control’s positive association with verbal thought percentage, which in turn was negatively associated with autonomic arousal. This is consistent with our model’s view that worry predicts lower autonomic arousal at high levels of effortful control because effortful control permits greater success in emphasizing verbal thought during worry. Nevertheless, we must emphasize the tentative nature of this support since all variables in the moderated mediation model were collected concurrently (Maxwell et al., 2011). Although our results are consistent with predictions of our model, prospective study designs, preferably with experimental manipulation of effortful control resources, are needed to support strong confidence in this aspect of our model.

Taken together with results from Vasey et al. (2016), the current findings suggest that there are pathological worriers and GAD patients who have the cognitive control capacity required to maintain a verbal mode of processing necessary, which is required to access negative reinforcement contingencies stemming from limiting activation of autonomic arousal. On the other hand, those who lack such capacity tend to experience fewer verbal thoughts during worry and consequently higher autonomic arousal as a consequence of worry. Such a pattern fits with the prerequisites for the negative reinforcement stemming from contrast avoidance.

We expected to find that the association between GAD symptom severity and percentage of images during worry was strongest when effortful control is low. However, the fact that we did not is perhaps not surprising given that another study using the same single item retrospective questionnaire failed to find it to be significantly associated with GAD symptoms. Specifically, another self-report study found no significant differences between GAD analogs and normal controls in the percentage of images reported during worry (Freeston et al., 1996). Furthermore, a laboratory study also found that controls and GAD patients did not differ in percentage of imagery during a worry period (Borkovec and Inz, 1990). Consistent with past findings, our study found that worry is predominantly verbal (65% verbal versus 25% imagery). Because of this low average percentage of imagery, it may be that statistical power to find effects is limited by range restriction. Future studies using mentation sampling during worry and relaxation periods may yield a more sensitive measure of variation in percentage of imagery during worry.

Also contrary to expectation, the interaction between worry/GAD symptom severity and effortful control was unrelated to self-reports of efforts to transform images into thoughts. One reason could be that accurate self-report of this construct rests on an untested assumption that this construct is consciously accessible to individuals (Sexton and Dugas, 2008). Furthermore, in validating the CAQ, Gosselin et al. (2002) reported that three of the five items from the CAQ-Transform scale loaded more highly on a different factor, suggesting that this construct is complex. As such, use of tasks such as mentation sampling may increase validity in future studies.

To this point we have focused on effortful control as a stable trait-like construct and its association to inter-individual differences in the level of autonomic arousal symptoms experienced during worry. However, effortful control capacity can vary within an individual (e.g., due to varying levels of cognitive load or stress). Given that, our integrative model thus also suggests the potential for intra-individual differences in autonomic arousal symptoms as a function of variations in a worrier’s ability to emphasize verbal worry. There are at least two possible paths to such differences. First, evidence suggests that constraining worry to such verbal modes of processing depletes cognitive resources (Leigh and Hirsch, 2011), which can lead to increased negative intrusions (Stokes and Hirsch, 2010) and promote further attention to threat (Williams et al., 2014). This suggests that worrying in a verbal manner may deplete the very resources needed to maintain such a verbal mode of processing. If so, even worriers and those with GAD who have high trait-level capacity for effortful control may experience increasing autonomic arousal symptoms during prolonged periods of worry, as their ability to suppress images and shift to a verbal mode of processing wanes. Second, during periods of other cognitive load or stress, such individuals may experience heightened autonomic arousal symptoms during bouts of worry because their capacity for effortful control and ability to constrain worry to a verbal mode has been depleted (Steinhauser et al., 2007). Furthermore, such heightened arousal may lead to an upward spiral in worry and autonomic arousal symptoms because perceptions of arousal appear to maintain worry among those high in GAD symptoms. An experimental study found that when asked to relax following a worry induction, GAD patients who were given false arousal feedback maintained their levels of worrying while those who were given false relaxation feedback decreased their levels of worrying (Andor et al., 2008). This suggests that during periods of prolonged stress worriers for whom worry usually functions to limit autonomic arousal symptoms may instead experience increased vulnerability to intrusive images and autonomic arousal symptoms as a result of stress-related effortful control resource depletion. Unfortunately, a test of this hypothesis awaits future research.

### Limitations

This study’s results should be considered in the context of several limitations. Although significant, it should be noted that the magnitude of variance accounted for by the interaction between GAD symptom severity and effortful control predicting autonomic arousal and, especially, percentage of thought during worry was small. However, in this regard it is important to recall that power to detect interactions is highest and such interactions will be strongest in samples that include many individuals who fall at the confluence of the extremes of the interacting dimensions in question (McClelland and Judd, 1993). In this case, it is most important for a sample to include as many individuals with high GAD symptom severity combined with either high or low effortful control. Because the current study utilized an unselected sample, in which most individuals inevitably fell toward the middle of the bivariate distribution defined by the interacting variables, the interaction term cannot account for much variance in the sample as a whole. Future studies should seek to oversample for such individuals to maximize statistical power to detect the interaction effect (McClelland and Judd, 1993).

With regard to the small amount of variance accounted for in predicting percentage of thoughts during worry, it is important to recall that the dependent variable was derived from a single-item measure. Single-item measures have been shown to have much poorer reliability than multi-item measures (Nunnally and Bernstein, 1994). Nevertheless, we thought such measures offered a reasonable starting point since they have been used successfully in other self-report studies using unselected samples especially for reports of thoughts during worry (e.g., Borkovec and Lyonfields, 1993; Freeston et al., 1996). Furthermore, that the single-item measure (i.e., percentage of thoughts) revealed the expected effect may be cause for optimism about the robustness of the effect. However, to increase the likelihood of replication of these findings, future studies should utilize more reliable and valid measures (e.g., thought sampling [Borkovec and Inz, 1990; Hirsch et al., 2012]).

This study was also limited because we did not obtain diagnostic information and cannot be sure how many members of our analog GAD group actually met DSM criteria for GAD. That said, we believe research on such samples is still useful, especially given that studies have shown that worry is continuously distributed in the population and that there are no clear boundaries between subclinical and clinical levels of worry and GAD symptoms (Ruscio et al., 2001; Olatunji et al., 2010). Moreover, our analog GAD group’s average PSWQ score (*M* = 67.09, *SD* = 9.96) is comparable to those reported for either analog GAD samples (*M* = 63.6, *SD* = 10.8) or clinical GAD samples (*M* = 67.2, *SD* = 9.2; Startup and Erickson, 2006). Nevertheless, replication in clinical GAD samples is needed to increase confidence that this model applies to a clinical population.

A further limitation was our exclusive reliance on self-reports. Future studies are needed to replicate these findings with objective measures of autonomic arousal and effortful control. However, with regard to such measures of autonomic arousal it is important to note that our model does not require that subjective and objective measures be concordant. In other words, the reinforcement mechanisms in the CognAv and ContrAv models should both operate even if they only involve subjective autonomic arousal. For example, in the case of the CognAv model, the negative reinforcement mechanism associated with a verbal mode of worrying would operate even if it were only linked to reductions in subjective experience of autonomic arousal. Similarly, it should be sufficient for the ContrAv Model if worry is linked to high levels of subjective arousal. Nevertheless, many of the studies of verbal versus imaginal processing of threat on which the CognAv Model is based used objective measures. Therefore, we certainly expect that our model can also account for heterogeneity in objective measures. Indeed, we recently completed an initial test of that hypothesis and found the self-reported GAD symptom severity (using the GADQ-IV) and effortful control (using the Adult Temperament Questionnaire – Effortful Control scale; Evans and Rothbart, 2007) interacted significantly in predicting mean HR during a baseline period (Free, 2017, Unpublished). Second, that study replaced self-reported effortful control with a measure of resting HRV, which provides a physiological measure of top–down control capacity (see e.g., Thayer et al., 2012). Results showed that like self-reports of effortful control, HRV significantly moderated the association between GAD symptom severity and autonomic arousal symptoms.

### Future Directions

In sum, this study’s findings provided a replication of the results reported by Vasey et al. (2016), showing that the Cognitive Control Model can account for the well-documented heterogeneity in level of autonomic arousal symptoms in worry and GAD. Furthermore, they serve to increase confidence that the model’s hypothesized effect of individual differences in cognitive control capacity on autonomic arousal does indeed occur even at very high levels of worry and GAD symptoms. Furthermore, the current findings extend prior work by offering initial support for the proposed mechanism of this model. Specifically, the percentage of verbal thoughts during worry varies as a function of level of effortful control capacity such that it is highest among worriers with high capacity for effortful control. Furthermore, it appears that the positive correlation between effortful control and verbal worry involves the same variance as the negative correlation between effortful control and autonomic arousal symptoms. Our test of moderated mediation thus supports, albeit preliminarily, the plausibility of our model’s prediction regarding the interplay between cognitive control capacity, predominance of verbal thought during worry, and autonomic arousal. Thus, the Cognitive Control Model can potentially reconcile the CognAv and the ContrAv models by showing how worry can serve either of the two models’ avoidant functions for worriers depending on their cognitive control capacity. In short, a worrier with high cognitive control capacity should have greater success in making and maintaining the shift to a verbal mode of threat processing posited by the CognAv Model, thereby limiting activation of autonomic arousal. In contrast, a worrier low in such capacity should have difficulty doing so, resulting in heightened autonomic arousal, thereby fostering avoidance of aversive contrasts due to unpredictable spikes in emotional arousal as postulated by the ContrAv Model. Thus, our findings have implications for better understanding the avoidant functions of worry in the etiology and maintenance of GAD. Although promising, however, a replication of these findings using multiple measures of worry, effortful control, and autonomic arousal at more comprehensive levels of analysis is needed to further foster confidence in our model. Specifically, future studies using a worry induction and monitoring the process of worry in real time (as opposed to retrospective self-report) would be an important advance. Similarly, use of EEG during relaxation and worry periods may yield objective measures of differing patterns of activation during verbal versus imagery-based worry. While subjective and objective autonomic arousal need not show concordance for our model to function as expected, because heterogeneity in the level of autonomic arousal is seen among worriers it is important for future work to evaluate the model in the context of psychophysiological measures of autonomic arousal. Finally, these findings should be replicated using behavioral measures of effortful control. These include behavioral measures (e.g., the Attention Network Test [ANT]) and physiological measures (e.g., resting HRV).

## Ethics Statement

All procedures performed in this study involving human participants were in accordance with the ethical standards of the Ohio State University Behavioral and Social Sciences Institutional Review Board and with the 1964 Helsinki declaration and its later amendments or comparable ethical standards. Informed consent was obtained from all individual participants included in the study.

## Author Contributions

GT: Designed the study, collected the data, performed analyses, and co-wrote the manuscript. MV: Designed the study, performed analyses, and co-wrote the manuscript.

## Conflict of Interest Statement

The authors declare that the research was conducted in the absence of any commercial or financial relationships that could be construed as a potential conflict of interest.

## Footnotes

1 http://www.afhayes.com

2 Applying the criteria used in Vasey et al. (2016) identified only 55 subjects. However, those criteria (i.e., meeting DSM-IV criteria for GAD based on the GADQ-IV and scoring 70 or higher on the PSWQ) were unusually very stringent and the resulting group was considerably more extreme on the PSWQ (*M* = 75.5 [*SD* = 3.1]) than typical analog GAD samples (*M* = 63.58 [*SD* = 10.8]) or even clinically diagnosed samples (*M* = 67.16 [*SD* = 9.2]; see Startup and Erickson, 2006).

3 Generalized Anxiety Disorder Questionnaire-IV scores were used as a measure of GAD symptom severity because the initial sample did not complete the WAQ.

4 This subsample overlaps with the analog sample in the current study. Specifically, the 46 subjects in the combined sample which were drawn from the current sample were all members of the analog sample drawn from the current sample (i.e., they comprised the most severe 38.3% of that analog sample).

5 All analyses were ran without the 18 cases with one or more missing scale scores. All significant results remained significant.

6 See the online supplement for a test of the PSWQ × EC and GADQ-IV × EC interaction predicting DASS-A scores. The interaction was significant in both cases.

7 See the online supplement for a test of the PSWQ × EC and GADQ-IV × EC interaction predicting percentage of thoughts during worry. The interactions were not significant and marginally significant respectively.

8 See the online supplement for a test of the PSWQ × EC and GADQ-IV × EC interaction predicting percentage of imagery during worry. The interactions were significant and not significant respectively.

9 See the online supplement for a test of the PSWQ × EC and GADQ-IV × EC interaction predicting efforts to transform imagery into thoughts. The interactions were not significant.

10 See the online supplement for a test of the indirect effect of GADQ-IV × EC interaction predicting DASS-A scores through percentage of thoughts during worry. The interaction was not significant but was in the direction expected.

11 Only a test of the indirect effect of percentage of thoughts was done because the other two candidate mediators were unrelated to the interaction.

12 The index of moderated mediation is an estimate of the slope of the line relating the indirect effect to the moderator (Hayes, 2015). If this index is statistically different from zero, there is significant moderated mediation.

13 We also tested an alternate model in which autonomic arousal interacted with effortful control to predict level of worry and percentage of verbal thoughts. The DASS-A × EC interaction was significant and produced a pattern consistent with expectation.
