# Supplementary material for: Heterogeneity in Autonomic Arousal Level in Perseverative Worry: The Role of Cognitive Control and Verbal Thought
Source: Front Hum Neurosci. 2017 Mar 13;11:108. doi: 10.3389/fnhum.2017.00108 (PMC5346585; doi:10.3389/fnhum.2017.00108)
Supplement: Supplementary file 1 [file Data_Sheet_1.docx]

Supplementary Material

Article title: Heterogeneity in Autonomic Arousal Level in Perseverative Worry: The Role of Cognitive Control and Verbal Thought

**Author names**: Gim Y. Toh and Michael W. Vasey

*** Correspondence:** Corresponding Author: vasey.1@osu.edu

# Supplementary Data

***Did EC interact with worry/GAD symptom severity to predict AA?***

As discussed in the main article, EC should have a moderating impact on AA. To test this, we asked if worry/GAD symptom severity (PSWQ/GADQ-IV score) predict AA differentially depending on level of EC?

***Results***

*PSWQ.* Table S1 shows, on average DASS-A scores were significantly positively predicted by the PSWQ and significantly negatively predicted by EC. The PSWQ x EC interaction was also significant. Examination of the region of significance revealed that PSWQ scores significantly positively predicted DASS-A scores for all levels of EC. That association was stronger when EC was low (simple slope [B] = .57, *p* < .001) versus high (B = .38, *p* < .001; see Figure S1). From the opposite viewpoint, EC scores significantly negatively predicted DASS-A scores for all levels of PSWQ but that association was stronger when PSWQ was high (B = -.45, *p* < .001) versus low (B = -.28, *p* < .001).

*GADQ-IV Score*. Table S1 shows the GADQ-IV score produced the same pattern of results as the PSWQ, with the GADQ-IV score, EC, and the GADQ-IV x EC interaction all achieving significance. GADQ-IV scores significantly positively predicted DASS-A scores across all levels of EC. However, that association was stronger when EC was low (B = .53, *p* < .001) versus high (B = .37, *p* < .001; see Figure S1). From the reverse perspective, EC scores significantly negatively predicted DASS-A scores across all levels of the GADQ-IV score. However, that association was stronger when the GADQ-IV score was high (B = -.38, *p* < .001) versus low (B = -.21, *p* < .001).

***Discussion***

As expected and consistent with the Cognitive Control Model, these results show that individual differences in EC moderated the link between worry and GAD symptom severity and AA symptoms such that this link is strongest when EC was low and weakest when EC was high.

***Did EC interact with worry/GAD symptom severity to predict percentage of thoughts?***

*PSWQ.* As shown in Table S2, the regression analysis revealed only a significant effect of PSWQ (B = 4.57, p < .001) in Step 1. Consistent with past research utilizing self-report questionnaire methods (Borkovec & Lyonfield, 2000; Freeston et al., 1998), the PSWQ was positively related to percentage of thoughts. Furthermore, the addition of the PSWQ x EC interaction in Step 2 did not produce a significant increment in$R^{2}$ in Step 2. However, even though the interaction was not significant, there was a trend in the expected direction where PSWQ scores significantly and more strongly positively predicted percentage of thoughts when EC was high (B = 5.32, p < .001) than when EC was low (B = 3.59, p = 0.022; see Figure S2). Examination of the region of significance revealed that PSWQ was significantly positively associated with percentage of thoughts for EC > -1.63.

*GADQ-IV.* As shown in Table S3, the regression analysis revealed only a significant effect of GADQ-IV (B = 4.46, p < .001) in Step 1. However, the addition of the GADQ-IV x EC interaction (B = 1.31, p = .099) approached significance in Step 2. GADQ-IV scores significantly positively predicted percentage of thoughts when EC was high (B = 6.06, p < .001), whereas this relationship only approached significance when EC was low (B = 2.47, p = 0.086; see Figure S2). Examination of the region of significance revealed that GADQ-IV was significantly positively associated with percentage of thoughts for EC > -1.23. From the reverse perspective, EC scores were marginally significantly and positively associated with percentage of thoughts when GADQ-IV was high (B = 2.64, p = 0.067). Examination of the region of significance revealed that the simple slope for EC was not significant for any value of GADQ-IV. Although as noted, the p-value approaches significance at high levels of GADQ-IV.

***Discussion***

It is interesting to note that although both the GAD symptom severity measures produced similar findings, the GADQ-IV produced weaker effects (i.e. approached significance) than the WAQ (as reported in the article). This could be because GADQ-IV was “not primarily developed to act as a measure of the complete range of severity but instead as a screening instrument” p. 348 (Rodebaugh et al., 2008). On the other hand, dimensional scoring may allow better assessment of symptom severity (Ruscio, 2002), such as in the WAQ. This is also consistent with the fact that EC did not interact significantly with the PSWQ in relation to percentage of thoughts in our study. While worry is the cardinal symptom of GAD, most worriers (i.e., those who score high on the PSWQ) do not meet criteria for GAD (Ruscio, 2002) and as such, GAD should not be equated with severe worry. Although there was no interaction between worry and EC to predict percentage of thoughts during worry, the pattern of the interaction was in the predicted direction. Therefore, it is likely that these effects may simply be weaker for non-GAD high worriers.

***Did EC moderate the indirect path between worry/GAD symptom severity and DASS-A that is mediated by percentage of verbal thoughts?***

*GADQ-IV.* Because the interaction between GADQ-IV and EC in predicting percentage of thoughts approached significance, we used a moderated mediation model to test whether the relationship between GADQ-IV and DASS-A was mediated by percentage of thoughts but conditional upon level of EC. However, as shown in Table S3, no significant moderated mediation was found for DASS-A (index of moderated mediation [Hayes, 2015]^1^ = -.0036; SE = .0027; LLCI = -.0112; ULCI = .0001). However, as shown in Table S4, note that the pattern of the effect was similar to when WAQ was used as a predictor and was in the direction expected, such that higher EC predicted higher percentage of thoughts, which in turn predicted lower DASS-A scores.

***Discussion***

There was some variability across measures of worry/GAD symptom severity. Whereas the interaction was not significant using the PSWQ, which is a measure of trait worry, the measure of GAD symptom severity yielded more encouraging results. While the interaction between GAD symptom severity and EC predicting AA was not significant, it approached significance with the pattern of the effect in the direction expected. Notably, as reported in the article, using a dimensional measure of GAD symptom severity, which increases the variability in the product term of the interaction, the interaction was significant. As discussed previously, these results suggest that these effects may simply be weaker for non-GAD high worriers and that the pattern may be stronger when using better measures of GAD symptom severity.

***Did EC interact with worry/GAD symptom severity to predict percentage of images?***

*PSWQ.* As shown in Table S5, the regression analysis revealed no significant effects of PSWQ or EC. However, the addition of the PSWQ x EC interaction (B = 1.14, p = .075) produced a marginally significant increment in$R^{2}$ in Step 2. As depicted in Figure S3, PSWQ was significantly negatively associated with percentage of images when EC was low (B = -2.47, p = .037), but not high (B = .57, p = 0.579). Examination of the region of significance revealed that higher levels of PSWQ were negatively associated with percentage of images for EC < -.66 SDs. However, it should be noted that the pattern of this interaction was such that the predicted percentage of images was similar for all combinations of high and low PSWQ and EC scores except for low PSWQ combined with low EC. The predicted percentage of images was similar among those scoring high on the PSWQ regardless of EC scores. Thus, viewed from the perspective of EC’s relationship with percentage of images, EC was not related to percentage of images when PSWQ was high (B = .50, p = .650). Examination of the region of significance revealed that the simple slope was significant only for values of PSWQ < -.21 SDs.

*GADQ-IV.* As shown in Table S5, the regression analysis revealed no significant effects of GADQ-IV, EC, or their interaction.

***Discussion***

While we did find that trait worry interacted with EC to predict percentage of images during worry, this interaction only approached significance, and thus should be interpreted with caution. Furthermore, we did not test a model of moderated mediation because the marginal interaction was the result of differences in EC among low worriers rather than high worriers. Thus, there was no support for our hypothesis that individuals with high trait worry with high EC experience lower percentage of images during worry compared to those with low EC. Additionally, while we expected to find an association between GAD symptom severity and percentage of images during worry, as mentioned in the article, the fact that we did not is consistent with another self-report study (Freeston et al. 1996) and a mentation sampling study (Borkovec & Inz, 1990).

***Did EC interact with worry/GAD symptom severity to predict efforts to transform images into thoughts?***

*PSWQ.* As Table S6 shows, on average CAQ-Thoughts scores were significantly positively predicted by PSWQ (B = .17, p < .001) and significantly negatively predicted by EC (B = -.22, p < .001). However, the PSWQ x EC interaction was not significant (B = -.03, p = .424).

*GADQ-IV.* Table S6 shows that the GADQ-IV produced the same pattern of results as the PSWQ, with GADQ-IV (B = .21, p < .001) and EC (B = -.19, p < .001) achieving significance but not their interaction (B = .01, p = .761).

***Discussion***

Unexpectedly, EC did not significantly moderate the link between worry/GAD symptom severity and efforts to transform thoughts into imagery. As discussed in the article, this construct may not be consciously accessible to individuals (Sexton & Dugas, 2008) and may be better assessed using other measures.

# Supplementary Figures and Tables

Table S1. Regression model testing PSWQ/GADQ-IV Score x EC predicting DASS-A

|  | ***Predictor: PSWQ*** | | |  | ***Predictor: GADQ-IV*** | | |  |
| --- | --- | --- | --- | --- | --- | --- | --- | --- |
| **Step** / Variable | **R^2^** / B | **ΔR^2^** / SE | *sr* | | **R^2^** / B | **ΔR^2^** / SE | *sr* | |
| ***Step 1*** | **.299^***^** | **-** |  | | **.374^***^** | **-** |  | |
| ***Step 2*** | **.303^*^** | **.004^*^** |  | | **.379^**^** | **.004^**^** |  | |
| Intercept | -.01 | .03 |  | | -.01 | .03 |  | |
| PSWQ/GADQ-IV | .33^***^ | .03 | .33^***^ | | .45^***^ | .03 | .43^***^ | |
| EC | -.36^***^ | .03 | -.35^***^ | | -.30^***^ | .03 | -.28^***^ | |
| PSWQ/GADQ-IV x EC | -.07^**^ | .03 | -.07^**^ | | -.07^**^ | .03 | -.07^***^ | |

Note: N = 926. ^***^*p* < .001; ^**^*p* < .01; ^*^*p* < .05; *^†^p* < .10.

Table S2. Regression model testing PSWQ Score x EC predicting Percentage of Thoughts

| **Step** / Variable |  | **R^2^** / B |  | **ΔR^2^** / SE |  | *sr* |
| --- | --- | --- | --- | --- | --- | --- |
| ***Step 1*** |  | **.029^***^** |  | **-** |  |  |
| ***Step 2*** |  | **.029** |  | **.001** |  |  |
| Intercept |  | 64.30^***^ |  | .86 |  |  |
| PSWQ |  | 4.57^***^ |  | .88 |  | .15^***^ |
| EC |  | .51 |  | .88 |  | .02 |
| PSWQ x EC |  | .65 |  | .85 |  | .03 |

Note: N = 926. ^***^*p* < .001; ^**^*p* < .01; ^*^*p* < .05; *^†^p* < .10.

Table S3. Moderated mediation results involving GADQ-IV x EC predicting DASS-A through Percentage of Thoughts

|  |  | ***Predictor: GADQ-IV*** | | | | |
| --- | --- | --- | --- | --- | --- | --- |
| **Step** / Variable |  | **R^2^** / B |  | SE |  | *sr* |
| **DV: Percentage of Thoughts** |  | **.0272^***^** |  |  |  |  |
| Intercept |  | 64.73^***^ |  | .90 |  |  |
| GADQ-IV |  | 4.32^***^ |  | .91 |  | .15^***^ |
| EC |  | .65 |  | .91 |  | .03 |
| GADQ-IV x EC |  | 1.35*^†^* |  | .82 |  | .05*^†^* |
| **DV: DASS-A** |  | **.382^***^** |  |  |  |  |
| Intercept |  | .12^**^ |  | .07 |  |  |
| Percentage of Thoughts |  | -.002^*^ |  | .001 |  | -.06^*^ |
| GADQ-IV |  | .46^***^ |  | .03 |  | .43^***^ |
| EC |  | -.29^***^ |  | .03 |  | -.27^***^ |
| GADQ-IV x EC |  | -.06^*^ |  | .01 |  | -.06^*^ |

Note: N = 926. ^***^*p* < .001; ^**^*p* < .01; ^*^*p* < .05; *^†^p* < .10.

Table S4. Bootstrapped estimates of the conditional indirect paths for the effect of GADQ-IV scores and EC on DASS-A

|  |  | Indirect effect |  | Bootstrapped SE |  | Bootstrapped LLCI |  | Bootstrapped ULCI |
| --- | --- | --- | --- | --- | --- | --- | --- | --- |
| Indirect effects of GADQ-IV at varying  levels of EC |  |  |  |  |  |  |  |  |
| 10th |  | -.0055 |  | .0043 |  | *-.0177* |  | *.002* |
| 25th |  | **-.0077** |  | **.0043** |  | **-.0184** |  | **-.0012** |
| 50th |  | **-.0099** |  | **.0050** |  | **-.0215** |  | **-.0016** |
| 75th |  | **-.0117** |  | **.0059** |  | **-.0259** |  | **-.0021** |
| 90th |  | **-.0135** |  | **.0070** |  | **-.0312** |  | **-.0024** |
| Indirect effect of EC at 90^th^ percentile of  GADQ-IV |  | **-.0059** |  | **.0040** |  | **-.0170** |  | **-.0004** |

Note: N = 926. Bootstrapped estimates are based on 5,000 samples. Significant effects appear in bold. LLCI = lower limit of confident interval; ULCI = upper limit of confident interval.

Table S5. Regression model testing PSWQ/GADQ-IV x EC predicting Percentage of Images

|  | ***Predictor: PSWQ*** | | |  | ***Predictor: GADQ-IV*** | | |  |
| --- | --- | --- | --- | --- | --- | --- | --- | --- |
| **Step** / Variable | **R^2^** / B | **ΔR^2^** / SE | *sr* | | **R^2^** / B | **ΔR^2^** / SE | *sr* | |
| ***Step 1*** | **.003** | **-** |  | | **.002** | **-** |  | |
| ***Step 2*** | **.007*^†^*** | **.003*^†^*** |  | | **.003** | **.001** |  | |
| Intercept | 25.96^***^ | .66 |  | | 25.69^***^ | .65 |  | |
| PSWQ/GADQ-IV | -.73 | .67 | -.04 | | .24 | .69 | .01 | |
| EC | -1.04 | .67 | -.05 | | -.79 | .69 | -.04 | |
| PSWQ/GADQ-IV x EC | 1.14*^†^* | .64 | .06*^†^* | | .59 | .62 | .03 | |

Note: N = 926. ^***^*p* < .001; ^**^*p* < .01; ^*^*p* < .05; *^†^p* < .10.

Table S6. Regression model testing PSWQ/GADQ-IV Score x EC predicting CAQ-transform

|  | ***Predictor: PSWQ*** | | | |  | | ***Predictor: GADQ-IV*** | | | |
| --- | --- | --- | --- | --- | --- | --- | --- | --- | --- | --- |
| **Step** / Variable | **R^2^** / B | **ΔR^2^** / SE | | *sr* | | | | **R^2^** / B | **ΔR^2^** / SE | *sr* |
| ***Step 1*** | **.095^***^** | **-** |  | | | **.105^***^** | | | **-** |  |
| ***Step 2*** | **.095** | **.001** |  | | | **.105** | | | **.000** |  |
| Intercept | -.01 | .03 |  | | | .01 | | | .03 |  |
| PSWQ/GADQ-IV | .17^***^ | .03 | .17^***^ | | | .21^***^ | | | .03 | .15^***^ |
| EC | -.22^***^ | .03 | .21^***^ | | | -.19^***^ | | | .03 | .03^***^ |
| PSWQ/GADQ-IV x EC | -.03 | .03 | -.03 | | | .01 | | | .01 | .01 |

Note: N = 926. ^***^*p* < .001; ^**^*p* < .01; ^*^*p* < .05; *^†^p* < .10.

## Supplementary Figures


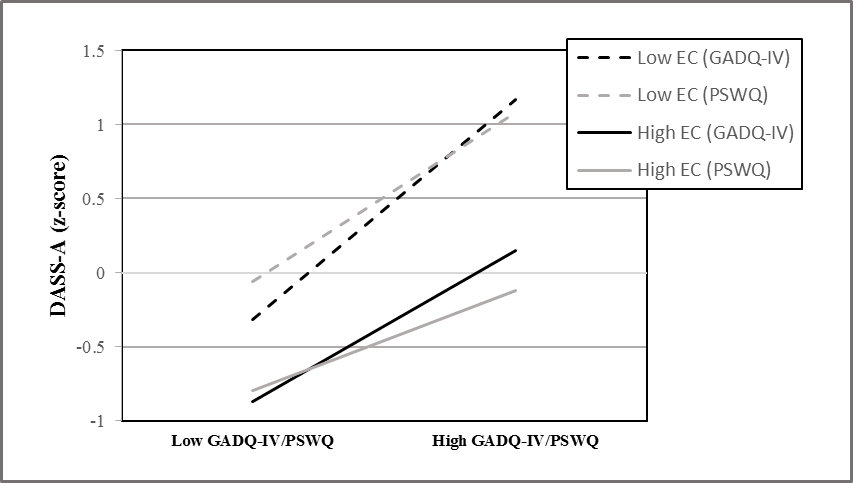


Figure S1. Graph of PSWQ/GADQ-IV x EC interaction predicting DASS-A.


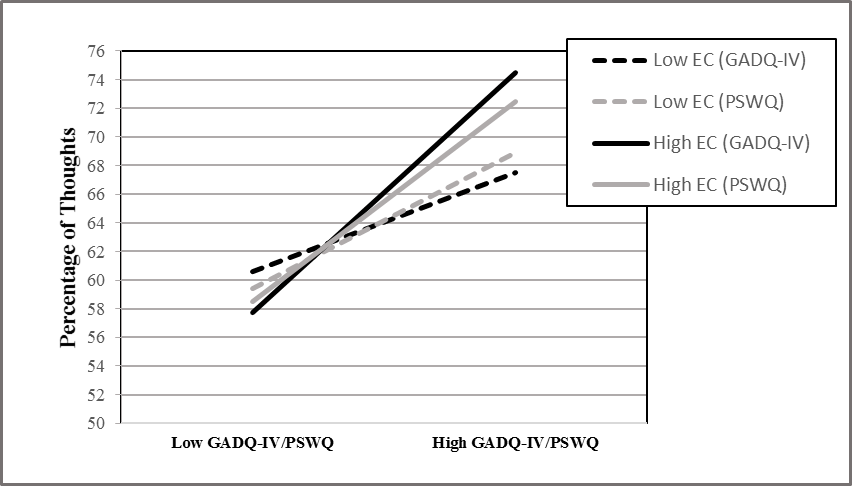


Figure S2. Graph of PSWQ/GADQ-IV x EC interaction predicting Percentage of Thoughts.


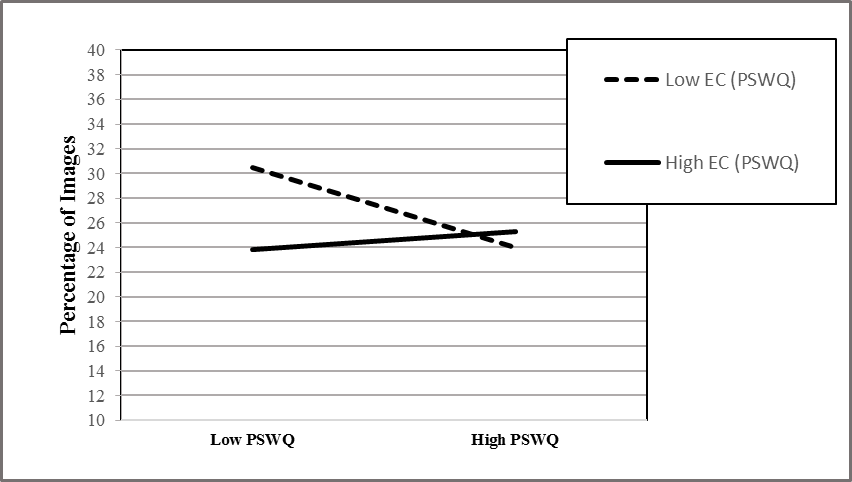


Figure S3. Graph of the PSWQ score x EC interaction predicting Percentage of Images.
